# Targeting Siderophore-Mediated Iron Uptake in *M. abscessus*: A New Strategy to Limit the Virulence of Non-Tuberculous Mycobacteria

**DOI:** 10.3390/pharmaceutics15020502

**Published:** 2023-02-02

**Authors:** Matteo Mori, Giovanni Stelitano, Giulia Cazzaniga, Arianna Gelain, Andrea Tresoldi, Mario Cocorullo, Martina Roversi, Laurent R. Chiarelli, Martina Tomaiuolo, Pietro Delre, Giuseppe F. Mangiatordi, Anna Griego, Loris Rizzello, Alberto Cassetta, Sonia Covaceuszach, Stefania Villa, Fiorella Meneghetti

**Affiliations:** 1 Department of Pharmaceutical Sciences, University of Milan, Via L. Mangiagalli 25, 20133 Milano, Italy; 2 Department of Biology and Biotechnology “Lazzaro Spallanzani”, University of Pavia, Via A. Ferrata 9, 27100 Pavia, Italy; 3 Institute of Crystallography, National Research Council, Trieste Outstation, Area Science Park–Basovizza, S.S.14-Km. 163.5, 34149 Trieste, Italy; 4 Institute of Crystallography, National Research Council, Via G. Amendola 122/o, 70126 Bari, Italy; 5 National Institute of Molecular Genetics (INGM), Via F. Sforza 35, 20122 Milano, Italy

**Keywords:** antimicrobial resistance, cystic fibrosis, drug design, grating-coupled interferometry (GCI), homology model, siderophores

## Abstract

Targeting pathogenic mechanisms, rather than essential processes, represents a very attractive approach for the development of new antimycobacterial drugs. In this context, iron acquisition routes have recently emerged as potentially druggable pathways. However, the importance of siderophore biosynthesis in the virulence and pathogenicity of *M. abscessus* (*Mab*) is still poorly understood. In this study, we investigated the Salicylate Synthase (SaS) of *Mab* as an innovative molecular target for the development of inhibitors of siderophore production. Notably, *Mab*-SaS does not have any counterpart in human cells, making it an interesting candidate for drug discovery. Starting from the analysis of the binding of a series of furan-based derivatives, previously identified by our group as inhibitors of MbtI from *M. tuberculosis* (*Mtb*), we successfully selected the lead compound **1**, exhibiting a strong activity against *Mab*-SaS (IC_50_ ≈ 5 µM). Computational studies characterized the key interactions between **1** and the enzyme, highlighting the important roles of Y387, G421, and K207, the latter being one of the residues involved in the first step of the catalytic reaction. These results support the hypothesis that 5-phenylfuran-2-carboxylic acids are also a promising class of *Mab*-SaS inhibitors, paving the way for the optimization and rational design of more potent derivatives.

## 1. Introduction

Non-tuberculous mycobacteria (NTM) are ubiquitous microorganisms that can act as facultative pathogens, causing severe infections in immunocompromised individuals and patients with congenital lung diseases, such as cystic fibrosis (CF) [1]. Among the more than 172 NTM species, *M. abscessus* (*Mab*) is becoming the most widespread and worrying pathogen for CF patients worldwide [2,3]. With an increasing incidence of about 20%, *Mab* has recently led to a significant growth in morbidity and mortality [4]. The standard treatment of this infection involves a very long regimen (up to two years of multidrug therapy with at least three antibiotics) that results in several issues for the patients, reducing the probability of success and dramatically increasing NTM resistance to antibiotics. Most notably, unsuccessful *Mab* eradication is considered an indication of lung transplantation. Hence, the development of new therapeutic strategies is necessary to improve clinical outcomes [5].

In recent years, several efforts have been made towards the development of alternative approaches against NTM infections, including host-directed therapy, repurposing of conventional drugs, use of ionic liquids, antimicrobial peptides, bacteriophages, and iron chelators [5]. Among non-traditional strategies, anti-virulence therapy, namely the use of compounds targeting pathways required for pathogenesis but dispensable for microbial growth, is emerging as a promising option [6]. Notably, this approach does not directly kill the pathogens. Instead, it is aimed at stopping them from attacking the host. Because of this, the potential drugs would not cause selective pressure, thus reducing the development of resistance [6].

**Figure 1:**
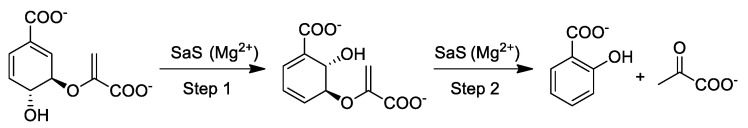
Scheme highlighting the reactions catalyzed by SaS in mycobacteria. In the first step, chorismate is converted to isochorismate, which is then transformed to salicylate, with the release of pyruvate.

Iron, an important co-factor for enzymes, virulence factors, and biofilm formation, is essential for mycobacteria to establish infection and proliferate. In detail, the ability of *Mab* to prevent phagosome maturation inside macrophages was shown to be dependent on iron uptake [7]. Hence, targeting the enzymes that mediate the acquisition of this ion could be an efficient strategy to inhibit bacterial growth. Mycobacteria have evolved different pathways to scavenge iron in the host, including the production of siderophores, whose biosynthesis has been extensively characterized in *Mtb* [8,9]. In this regard, it has been shown that mutations in the enzymes involved in this process can lead to lower growth of *Mtb* in macrophages and decreased virulence in mice [10,11]. The possibility of effectively targeting these enzymes with small-molecule inhibitors has been demonstrated in many studies [12,13]. The Salicylate Synthase (SaS) from *Mtb* (MbtI) is the first enzyme involved in siderophore production; it catalyzes the two-step conversion of chorismate to salicylate, via isochorismate as an intermediate, acting as both an isomerase and a lyase (Figure 1). Therefore, taking advantage of our experience in the inhibition of MbtI [14,15,16,17,18,19], we explored the possibility of developing potent enzymatic inhibitors of *Mab*-SaS.

Considering the high percentage of sequence identity between MbtI and the corresponding *Mab* enzyme (i.e., 63%), and, especially, the conservation of their active sites, we decided to test on *Mab*-SaS our library of previously discovered MbtI inhibitors (about 100 published and unpublished furan-based derivatives). Among them, three analogs, namely 5-(2,4-bis(trifluoromethyl)phenyl)furan-2-carboxylic acid (**1**), 5-(2-amino-4-nitrophenyl)furan-2-carboxylic acid (**2**), and 5-(3,5-bis(trifluoromethyl)phenyl)furan-2-carboxylic acid (**3**), emerged as promising candidates (Table 1). Notably, the low toxicity of this class of compounds has been previously assessed against human MRC-5 fibroblasts and blood cells [16,19].

In this work, we evaluated the activity and affinity of this collection of analogs against *Mab*-SaS by a comprehensive biochemical and biophysical characterization. Moreover, a computational investigation allowed us to describe the molecular basis of the inhibitory activity of the selected inhibitors. The gathered data will hopefully lead to relevant therapeutic advances in the treatment of *Mab* infections.

## 2. Materials and Methods

### 2.1. Synthesis and Characterization

The results of the screening of our in-house library prompted us to select compounds **1**–**3** for further evaluation; therefore, they were re-prepared according to published procedures [16,18]. Briefly, **1** and **3** were obtained by a Suzuki reaction between methyl 5-bromofuran-2-carboxylate and the appropriate phenylboronic acid, followed by a hydrolysis in basic conditions. Similarly, **2** was synthesized by a Suzuki coupling between (5-(methoxycarbonyl)furan-2-yl)boronic acid and 2-bromo-5-nitroaniline, followed by a base-catalyzed hydrolysis of the ester moiety. The intermediates and final compounds were purified by traditional methods and characterized by ^1^H NMR, ^13^C NMR, FT-IR, and mass spectrometry. Data were consistent with the literature (see Supplementary Materials).

### 2.2. Mab-SaS Expression, Purification, and Characterization

The MAB_2245 gene (*Mab* ATCC_19977), encoding the *Mab*-SaS, was cloned into the BamHI-HindIII sites of the pET-28a vector (GenScript, Piscataway, NJ, USA), using the InFusion cloning Kit (Takara Bio Europe SAS, Saint-Germain-en-Laye, France) and the following primers: Forward 5′-ATGGGTCGCGGATCCGAAAACCTGTATTTTCAGGGCGTGTCGAAGATGAGTGCG-3′; Reverse 5′-TGCGGCCGCAAGCTTCTATGCCTCGCGCTTG-3′. Primers were designed to insert the sequence recognized by TEV protease at the N-terminus, for the removal of the His-Tag. The protein was expressed in *E. coli* BL21(DE3) cells, grown to an OD_600_ of 0.8, and then induced by 1 mM IPTG at 25 °C overnight. The protein was purified to homogeneity by Immobilized Metal Affinity Chromatography (IMAC) and Gel Filtration (GF). The activity of the enzyme was determined at 37 °C by a fluorimetric assay, as previously reported [17]. Briefly, the assays were performed in 400 μL of 50 mM Hepes pH 7.5 containing 5 mM MgCl_2_, and 1–2 μM *Mab*-SaS, started by the addition of chorismic acid and monitored using a PerkinElmer LS3 fluorimeter (Ex. λ = 305 nm, Em. λ = 420 nm; PerkinElmer, Waltham, MA, USA). Steady-state kinetic analysis was performed measuring the enzymatic activity at different concentrations (10–500 μM) of chorismic acid. All measurements were performed in triplicate, and K_M_ and k_cat_ were determined using the Michaelis-Menten equation, with GraphPad Prism 8 software (GraphPad Software, San Diego, CA, USA).

### 2.3. Biological Tests

#### 2.3.1. *Mab*-SaS Activity Dependence on Magnesium Concentration

The effect of magnesium concentration on the enzymatic activity of *Mab*-SaS was determined, as above, at subsaturating concentrations of chorismic acid (50 μM), in the presence of variable concentrations of MgCl_2_ (0.25–100 mM) (Figure S1).

#### 2.3.2. *Mab*-SaS Inhibition Assays

The selected furan-based compounds were screened against *Mab*-SaS enzymatic activity at 100 µM (dissolved in a solution containing 10% DMSO). Assays were performed at subsaturating concentrations of chorismic acid (50 μM), as above. For the most promising compounds, the IC_50_ value was also determined.

#### 2.3.3. Pan Assay Interference Compound (PAIN) Analysis

In our previous works, we extensively demonstrated that the members of our furan-based library did not act as PAINS. To further verify the reliability of the inhibition results against *Mab*-SaS, we determined the IC_50_ of compound **1** in the presence of 0.1 mg/mL bovine serum albumin (BSA) or in the presence of 0.01% (*v*/*v*) Triton X-100 as a detergent [14,15,16,17,18]. Moreover, to exclude an unspecific binding to cysteine residues, we also tested the IC_50_ with 100 μM of 1,4-dithio-DL-threitol (DTT).

#### 2.3.4. Siderophore Production Assay

The production of siderophores was assayed in *M. bovis* BCG cultures by the isolation of mycobactins and by the Universal CAS liquid assay [20]. Briefly, *M. bovis* cells were firstly grown in 7H9 medium, and subsequently subcultured in chelated Sauton’s medium. The culture was then diluted 1:1000 in chelated Sauton’s medium in the presence of different concentrations of compound **1**, and incubated for 15 days at 37 °C. To isolate the mycobactins, cells were centrifuged, and pellets were extracted overnight in EtOH. 0.1 M FeCl_3_ in EtOH was added until no further change in color was observed, and the solution was incubated at room temperature for 1 h. Mycobactins were then extracted in CHCl_3_, washed with water 3 times to remove the excess of iron, and dried by evaporation; the residue was dissolved in MeOH. The concentration of mycobactins was determined by measuring the absorbance at 450 nm (1% solution of mycobactins has an absorbance of 42.8). For the Universal CAS assay, the supernatant of cell cultures (100 μL) was mixed with 100 μL of CAS solution in a 96-well plate, incubated for 10 min at room temperature; the absorbance at 630 nm was measured, and siderophore units were calculated with the following equation:

$$\frac{A_{r}{-A}_{s}}{A_{r}}\times 100$$

A_r_: absorbance at 630 nm of the blank medium with the CAS solution; A_s_: absorbance of the culture supernatants with the CAS solution.

#### 2.3.5. Minimal Inhibitory Concentration (MIC) Evaluation by Resazurin Assay

Exponentially growing primary cultures were diluted 1:10 in iron-depleted Sauton’s medium enriched with 10% OACD. The secondary *Mab* culture growing in iron-limiting conditions was then diluted to OD_600_ 0.005 using iron-depleted Sauton’s medium. Each well of the 96-well plate was filled with 100 μL of the diluted cell suspension. For each plate, the first four wells of the first row were filled with the cellular suspension and no drug was added (negative control). The following four wells were filled with the cellular suspension and treated with the vehicle volume. The last four wells of the first row were filled with the cellular suspension and treated with 1 mg/mL Streptomycin (positive control). Only the first well of the other rows was filled with 200 μL of cell suspension. In this well, the highest concentration of compound **1** (500 µM) was added and then two-fold serially diluted. After 24 h of incubation at 37 °C, 10 μL/well of 0.01% resazurin was added. The plate was re-incubated at 37 °C for 24 h and then read out. Pink wells indicated bacterial viability, whereas wells with an unchanged blue color indicated cidality. The MIC was counted as the lowest compound concentration causing cidality.

### 2.4. Binding Analysis

The binding affinity and ligand efficiency of the furan-based library was evaluated by Grating-Coupled Interferometry (GCI). The analyses were performed by the Creoptix WAVE system (Malvern Panalytical, Malvern, UK). Borate buffer (100 mM sodium borate pH 9.0, 1 M NaCl) was used for chip conditioning. Experiments were carried out by reversibly capturing His-tagged *Mab*-SaS (50 μg/mL in running buffer, i.e., PBS, 0.05% Tween 20, 0.5% DMSO) on 4PCP-NTA WAVE chips, according to the manufacturer’s instructions, at a density of 3000 pg/mm^2^.

Regeneration-free injections of a 1:2 dilution series in the 500–3 μM range for each compound of the furan-based library in running buffer were performed at 25 °C, using a flow rate of 100 μL/min.

A dimethyl sulfoxide (DMSO) calibration curve was used for bulk correction, and blank injections for double referencing. Data were analyzed and corrected using the Creoptix WAVE control software (correction applied: X and Y offset; DMSO calibration; double referencing). The processed data were fitted using the “1:1” kinetic model of the WAVE control software. All the experiments were performed in triplicate. Descriptive Statistical Analysis, as implemented in Microsoft Office Excel 10 (Microsoft Corporation, Redmond, WA, USA), was employed to calculate the mean of the obtained values of k_on_, k_off_, and K_D_ as a measure of central tendency; standard deviations of the former values were computed as a measure of dispersion.

### 2.5. Computational Details

#### 2.5.1. Homology Modelling

The homology model of *Mab*-SaS was built based on the published X-ray data of the *Mtb* congener (MbtI) [14]. *Mab*-SaS and MbtI share a significant sequence identity (63%), thus supporting the robustness of the developed model. Moreover, the X-ray structure used as a template was published in complex with 5-(3-cyanophenyl)furan-2-carboxylate, a ligand belonging to our series of furan derivatives, suggesting with a high degree of confidence that the binding site conformation of the developed model is that responsible for the molecular recognition of this class of compounds. Such a condition is a crucial prerequisite for running reliable docking simulations, as observed for other targets [21]. Prime [22], available from the Schrödinger suite, was used as software. More specifically, all the default settings were used, and the cognate ligand of the employed template was included in the final model. Finally, the obtained homology model was pre-treated with the protein preparation module available from the Schrödinger suite [22,23,24,25]. This tool allows to: (i) add missing hydrogen atoms; (ii) determine the optimal protonation and tautomerization states of the residues; (iii) fix the orientation of any misoriented groups, and (iv) perform a final energy minimization.

#### 2.5.2. Molecular Docking Simulations

Compounds **1**, **2**, **3**, and chorismate were docked in the binding site of the *Mab*-SaS homology model, generated as previously described. All the ligands were prepared using LigPrep [26], available from the Schrödinger suite, which allows the generation of all the possible tautomers and ionization states at a pH value of 7.0 ± 2.0. Docking simulations were performed by using Grid-based ligand docking with energetics (GLIDE) as a software program [27,28]. More specifically, the docking simulations were performed allowing full flexibility for the ligands and holding fixed the remaining part of the protein. Furthermore, the standard precision (SP) protocol and the default Force Field OPLS_2005 were used [29]. In detail, to appropriately explore the conformational space of the ligands during the simulations, the number of poses generated in the initial phase of docking were increased from a default setting of 5000 to 50,000, while the number of poses per ligand maintained for energy minimization ranged from a default setting of 400 to 4000. A cubic grid centered on the cognate ligand with an edge of 10.0 Å for the inner box and 22.3 Å for the outer box was employed. To challenge the robustness of the protocol, the cognate ligand was redocked into its corresponding binding site. To our delight, the top-scored docking pose was in full agreement with the X-ray data available in the template structure and kept in the developed homology model, as testified by the corresponding root mean square deviation (RMSD) value computed taking into account all the heavy atoms and equal to 0.04 Å. Considering that all the docked ligands shared a common scaffold with the cognate ligand, the conformational space explored during the docking simulations was restricted so that only poses matching the coordinates of the common substructure (RMSD tolerance equal to 1.0 Å) were generated.

#### 2.5.3. MM-GBSA Calculations

All the obtained top-scored docking complexes were subjected to molecular mechanics/generalized Born surface area calculations (MM-GBSA) [30] to compute the corresponding ligand-protein binding free energies (hereinafter referred to as ∆G). The calculation was performed using Prime [22]. Flexibility was allowed for all the residues having at least one atom within a distance of 4 Å from the ligand, and the “minimize side chain only” option was used for sampling.

## 3. Results

### 3.1. Biochemical Analyses

Our goal was to identify inhibitors of *Mab*-SaS among members of a library of 5-phenylfuran-2-carboxylic acid derivatives, and to gather preliminary data for the development of drug candidates targeting iron uptake in NTM. To this end, we produced the enzyme in recombinant form to assay the compounds belonging to our series of MbtI inhibitors. The recombinant *Mab*-SaS was demonstrated to be catalytically active; kinetic constants (k_cat_ 3.9 ± 0.2 min^−1^; K_M_ 0.058 ± 0.009 mM) were very similar to those of other mycobacterial enzymes (Figure S2). Moreover, the *Mab* enzyme, similarly to other mycobacterial SaSs, was found to be dependent on magnesium ions for its activity. In detail, the activity of *Mab*-SaS was negligible in the absence of MgCl_2_, increased reaching its maximum at 2 mM, and decreased at higher concentrations; a similar behavior was found for the *Mtb*-SaS [17]. Contextually, the purified protein was confirmed to be suitable for the screening of potential inhibitors. Therefore, we assayed about 100 derivatives, and we found that some of them exhibited strong activity against *Mab*-SaS. The effect of the selected compounds was measured at 100 μM as a percentage of residual enzymatic activity. The values of the three most promising inhibitors (**1**–**3**) are reported in Table 1, along with their IC_50_.

The most potent inhibitor **1** was selected as a suitable candidate to deepen our biological investigations. To provide evidence to explain the role of the Mg^2+^ co-factor on the activity and inhibition of *Mab*-SaS, the inhibitory activity of **1** was also determined at increasing concentrations of Mg^2+^, showing that the metal did not influence the binding of the compound to the target.

**Figure 2:**
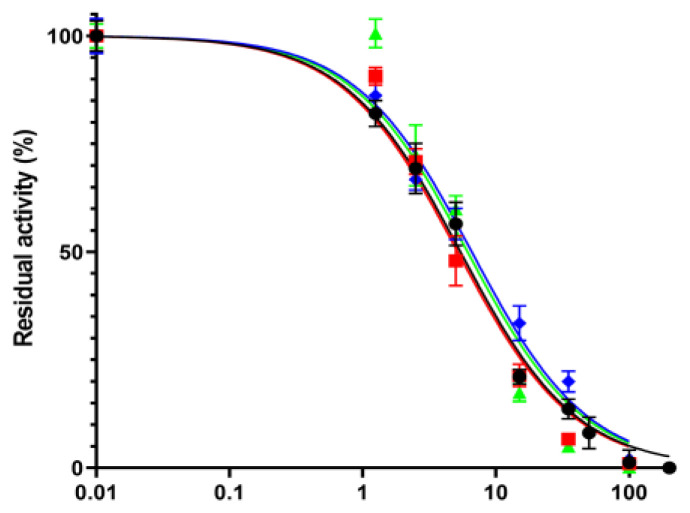
Inhibitor **1** does not behave as a PAIN compound. The IC_50_ value determined in the absence of any additive (black) was consistent with those measured in the presence of 1 mg/mL BSA (red) or 0.01% Triton X-100 (green), confirming that the compound does not induce aggregation, and in the presence of 100 μM DTT (blue), excluding an interaction of **1** with the cysteine residues of *Mab*-SaS.

Compound **1** was also subjected to different analyses to ascertain that it did not act as a PAIN compound [31]. The performed tests confirmed that **1** was not an interferent; as shown in Figure 2, its IC_50_ value in the presence of BSA (5.2 ± 0.68 μM) or Triton X-100 (6.5 ± 0.56 μM) did not differ significantly from the previously reported value (Table 1). Moreover, the presence of 100 μM DTT in the reaction mixture did not alter the IC_50_ of **1** (6.1 ± 1.42 μM), excluding a promiscuous inhibition of the enzyme through covalent reaction of the compound with cysteine residues.

### 3.2. Binding Analysis

**Figure 3:**
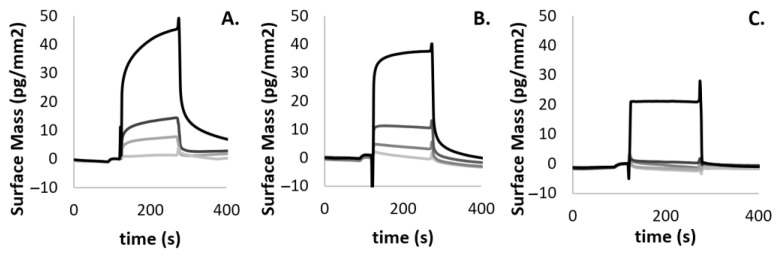
Quantitative binding kinetics of *Mab*-SaS vs. **1** (**A**), **2** (**B**), and **3** (**C**), measured by GCI. All curves were blank subtracted. Experiments were performed in triplicate. All the quality assessments (i.e., χ^2^ shown in Table 1, parameter errors, and residual plots were acceptable; the sensorgrams had sufficient curvatures and the kinetic constant k_off_ were within the measurable range) were fulfilled. Descriptive Statistical Analysis was employed to calculate the mean of the obtained values of k_on_, k_off_, and K_D_ as a measure of central tendency; standard deviations of the former values were computed as a measure of dispersion.

To understand whether and how *Mab*-SaS was able to discriminate between related molecules, accurate quantification of binding affinities and kinetics was performed. In detail, the interaction between *Mab*-SaS and the furan-based MbtI inhibitors was characterized by GCI. This technique was selected because it combines the highest sensitivity with the broadest sample compatibility. Its application is suitable for a wide range of pharmacological targets and ligands (especially very-low-molecular-weight compounds) and can reliably resolve very weak binders with off-rates even faster than 5 s^−1^ [32]. The most promising compounds yielded dissociation constants (K_D_) ranging from ≈ 6.11 to 67.50 μM (Table 2 and Figure 3).

As shown in Table 2, one of the selected compounds was found to be provided with a potent activity (**1**, K_D_ ≈ 6 μM), another displayed a remarkable activity (**2**, K_D_ ≈ 13 μM), while the latter revealed a modest activity (**3**, K_D_ ≈ 68 μM).

### 3.3. Microbiological Tests

**Figure 4:**
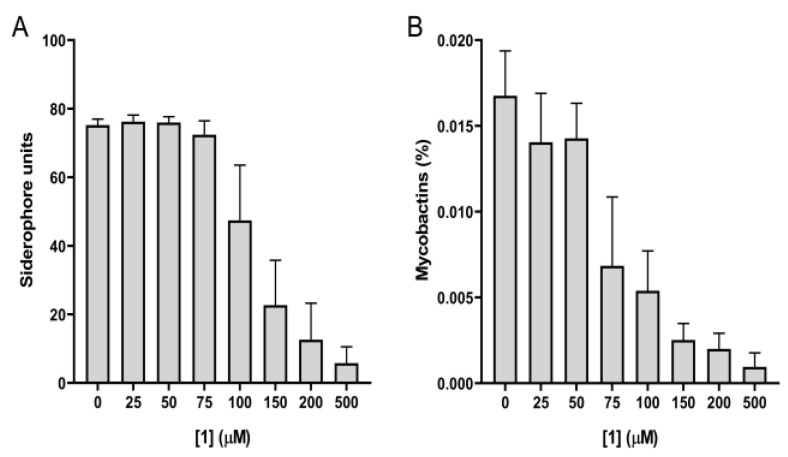
Compound **1** inhibits the production of siderophores and mycobactins by *M. bovis* BCG in iron-limiting conditions. (**A**) Siderophore units in culture media of cells grown in the presence of different concentrations of **1**, determined by the Universal CAS assay. (**B**) Determination of mycobactins extracted and measured in the above-mentioned cells. Data are mean ± SD of three replicates.

The antimycobacterial activity of **1** was tested on *Mab*, in iron-limiting conditions, using the chelated Sauton’s medium. The compound did not show any effect up to a concentration of 500 μM. Considering its lack of activity on *Mab*, the ability of **1** to inhibit the production of siderophores was tested on *M. bovis* BCG, against which it exhibited antimycobacterial activity at 500 μM [16]. As shown in Figure 4, the concentration of siderophores and mycobactins dropped as the compound concentration increased, confirming that compound **1** interfered with their production.

### 3.4. Molecular Modelling

**Figure 5:**
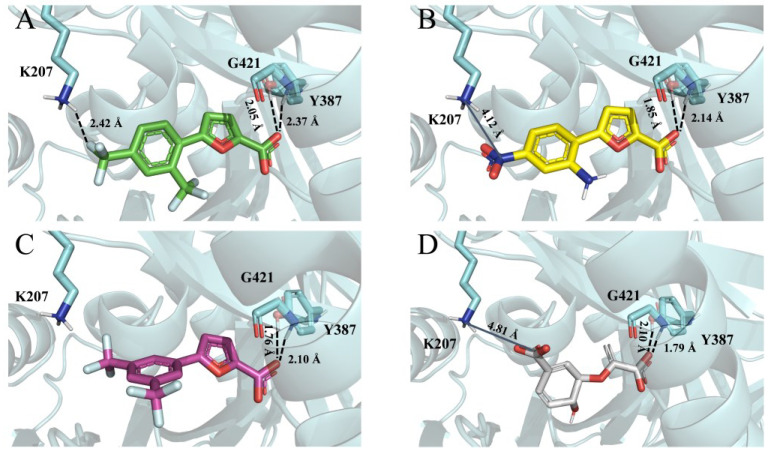
Top-scored docking poses of **1** (**A**), **2** (**B**), **3** (**C**), and chorismate (**D**) within the binding site of the developed *Mab*-SaS homology model. Ligands and important residues are rendered as sticks, while the protein as cartoon. H-bond and salt-bridge interactions are depicted by a dotted black line and a blue line, respectively. For the sake of clarity, only polar hydrogen atoms are shown. Notice that the fluorine atoms belonging to the trifluoromethyl substituents of **1** and **3** are depicted as cyan sticks.

As expected, the binding mode of the cognate ligand, observed in the template X-ray structure of MbtI (PDB code: 6ZA4) [17], was substantially retained in the *Mab*-Sas model: all the compounds were predicted to form interactions with two conserved residues of the binding pocket, namely Y387 (Y385 in MbtI) and G421 (G419 in MbtI). More specifically, the carboxylate moiety established two well-oriented H-bond interactions with the side chain of Y387 and the backbone of G421. Notably, the analog that was empirically proven to be responsible for the increased affinity (i.e., **1**) participated in an extra interaction with the side chain of K207 through its trifluoromethyl substituent (Figure 5A). A salt bridge interaction with the same residue was expected to be caused by the presence of a nitro group in the same location (**2**, Figure 5B). Visual assessment of the top-scored docking pose of **3** revealed no additional interactions (Figure 5C). Remarkably, these slightly different docking poses were responsible for diverse MM-GBSA binding free energies (∆G—see the experimental section for methodological details). In detail, the obtained values agreed with the experimentally measured affinity data, being the ∆G returned by **1** (−47.27 kcal/mol) better than those computed for **2** (−44.76 kcal/mol) and **3** (−44.29 kcal/mol). Finally, the same docking protocol was applied to chorismate. In agreement with the available experimental data (K_m_ = 58 µM), the computed binding free energy (−38.80 kcal/mol) as well as the obtained docking pose (Figure 5D) were close to those returned by the other investigated compounds, further corroborating the robustness of the adopted docking procedure. More specifically, chorismate was predicted to establish a salt-bridge interaction with K207 (as compound **2**) and two H-bond interactions already observed for all the other compounds, namely with the side chain of Y387 and the backbone of G421.

## 4. Discussion

*Mab* represents a growing and significant global health concern. This opportunistic pathogen can cause chronic pulmonary infections, especially among individuals with structural lung diseases, such as CF [3]. Current treatments require long periods of hospitalization, and the outcomes are often disappointing. Moreover, the effectiveness of the available drugs is severely compromised by the insurgence of multidrug resistance. Therefore, novel drugs acting on new molecular targets are urgently needed.

Among the most innovative approaches to fight bacterial infections, anti-virulence therapy appears to be very promising [6]. This strategy targets metabolic pathways that are crucial for the pathogenesis, while being non-essential for microbial growth [33]. With this approach, it is possible to prevent the establishment of the infection without exerting a selective pressure on the microorganism. This translates into a significant reduction of resistance mechanisms being developed during the therapy. In this context, iron is known to play a fundamental role in microbial physiology, especially during pathogenic processes. In mycobacterial infections, the survival of the *bacilli* in the host relies on the efficient uptake of this metal, which acts as a co-factor in several biological processes. Therefore, there is a growing interest in targeting the pathways involved in its acquisition [34,35,36]. In detail, the production of siderophores (mycobactins and carboxymycobactins) has been shown to be of crucial importance; hence, proteins involved in their assembly, transport, and functionality have been proposed as potential targets [37,38,39,40,41,42]. Moreover, siderophores have been shown to prevent phagosome maturation inside macrophages and induce hypoxia-like toxicity in host cells [7,43]. A number of enzymes are involved in their biosynthesis [9]; among them, SaS (known as MbtI in *Mtb*) has sparked the interest of the scientific community because it catalyzes the first step of the pathway. Moreover, it has no homologs in humans, which reduces the likelihood of off-target events.

In light of these considerations, there is a great interest in deciphering the mechanisms of siderophore assembly in *Mab*, with the goal of targeting selected steps for the development of new drugs. Despite these mechanisms being still poorly characterized in NTM, many research works have been focused on gathering new data, especially in the last years [44,45,46]. These preliminary studies seem to support the role of siderophores during *Mab* infection and suggest a conservation of the enzymes involved in their production.

Therefore, taking advantage of our expertise in the development of MbtI inhibitors [14,15,16,17,18,19], we performed functional and structural studies on the SaS encoded by *Mab*, investigating, for the first time, its potential role as a pharmacological target. In this work, we identified three enzymatic inhibitors that could represent the starting point for the design of more potent analogs. Our approach, integrating modelling data with biochemical and binding analyses, revealed the key determinants of *Mab*-SaS inhibition, paving the way to the development of novel therapeutics capable of hindering the virulence and pathogenesis of this *bacillus*.

We approached this study by developing an optimized procedure for the recombinant production and purification of wild-type *Mab*-SaS. Then, considering the high degree of conservation of this enzyme among mycobacteria, we used our library of furan-based compounds, previously developed as MbtI inhibitors, to select potential ligands. This preliminary screening led to the identification of **1** as the first potent inhibitor of *Mab*-Sas, supporting the use of this scaffold for the targeting of this enzyme in phylogenetically related bacteria. Moreover, the investigation of the inhibitory mechanism of *Mab*-SaS produced compelling experimental data that helped to clarify its functional properties, which are currently poorly known.

Compound **1** displayed the highest inhibition at 100 μM (98.8%), and its half-maximal inhibitory concentration (IC_50_) was determined to be ~5 μM (Table 1), which was lower than that measured for MbtI (~13 μM). The binding affinity of **1** was measured using GCI, resulting in a K_D_ value of about 6 μM. These results revealed that **1**, which exhibited the most promising inhibitory capabilities, can be considered the lead compound of this chemical class and, more importantly, the first inhibitor of *Mab*-SaS.

Docking simulations complemented the experimental findings by providing a molecular rationale behind the observed affinity data. To elucidate the molecular determinants of the interaction between **1** and *Mab*-SaS, we docked the selected inhibitors **1**–**3** into a homology model, built on the basis of the experimental coordinates of MbtI (PDB code: 6ZA4). Compound **1** showed electrostatic and hydrogen-bonding interactions with the target enzyme, establishing a contact with the side chain of K207. This additional interaction was probably responsible for the higher activity of **1** compared to its analogs. Most notably, the information emerging from the structure-based study indicated that the decoration of the phenyl moiety with H-bond acceptors in the *para* position with respect to the furan ring could be a valuable strategy to maximize the affinity of the compounds for the target.

Further experimental data were gathered to explore the binding mechanism of the ligand. To this end, the activity of **1** was assayed at different concentrations of Mg^2+^ to determine the role of the co-factor in the enzyme-inhibitor interaction. Our results showed that the metal did not strongly influence the binding of the compound to the target. However, at a very high concentration of Mg^2+^, the inhibitory effect of **1** was slightly reduced. Thus, it can be inferred that **1** might behave as a competitive inhibitor, preventing the binding of Mg^2+^. Accordingly, the compound would become somewhat less effective if the experimental conditions involved a high concentration of the metal. Incidentally, *Mab*-SaS was found to be completely non-functional in the absence of Mg^2+^, reaching its maximal activity at 2 mM of MgCl_2_. This observation confirmed that the ion acts as an essential co-factor, although the affinity seems rather moderate. Interestingly, these results are analogous to those previously obtained by our group for MbtI [17].

Based on these data, and by analogy to *Mtb*, we could hypothesize that a dysfunctional SaS in *Mab* could impair the production of siderophores, leading to an intracellular iron-deficiency state that would result in the death of the pathogen. Therefore, SaS appears to be one of the most promising antimycobacterial targets for potential therapeutic development. Notably, our computational study led to the construction of a homology model that will greatly assist in guiding future structure–activity relationship studies towards the generation of improved analogs with enhanced activity. Moreover, our results contributed to increase the knowledge of the siderophore production pathway in *Mab*, which is central to understand the mechanism of iron acquisition within the host and its role in pathogenesis. Further investigations are warranted to confirm the essential role of SaS, and siderophore production in general, in the growth of *Mab* in iron-limiting conditions, despite recent studies have already suggested this to be the case [44].

Our work has shown that 5-phenylfuran-2-carboxylic acids represent a new and promising chemical class of *Mab*-SaS inhibitors. These easy-to-prepare compounds are particularly interesting from a medicinal chemistry point of view because they can be conveniently adapted to obtain a variety of derivatives. Their rational modification, based on computational studies, will hopefully lead to the identification of more potent, optimized candidates, which will be developed into drugs to improve the clinical outcomes of *Mab* infections.

## 5. Conclusions

In this work, we reported the first study focused on *Mab*-SaS, a promising target for the development of an innovative antimycobacterial therapy aimed at reducing the pathogenicity of *Mab* without potential toxicity or resistance issues. The screening of a library of furan-based MbtI inhibitors led to the identification of compound **1**, which showed potent inhibition of the target enzyme. The analysis of its binding mode, based on molecular docking simulations, provided crucial information on its key interactions and suggested potential sites of modification. These findings will not only allow the development of a new pharmacophore model, but also constitute the basis for virtual screening approaches. These strategies will enable the discovery of novel, structurally diverse molecules, potentially capable of targeting mycobacteria in an effective and safe way.

## Figures and Tables

**Table 1:** In vitro inhibitory activities of compounds **1**–**3** against *Mab*-SaS.

Code	Structure	%RA *	IC_50_ (μM)
**1**	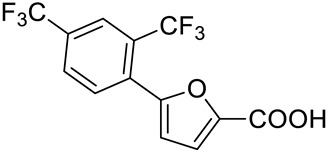	1.2 ± 2.8	5.3 ± 1.5
**2**	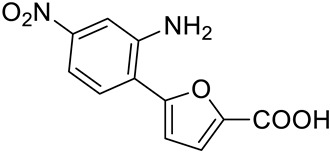	2.3 ± 0.7	23.6 ± 1.8
**3**	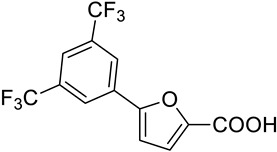	15.6 ± 2.5	29.5 ± 1.6

* Inhibitory effects are expressed as a percentage of the residual enzymatic activity (RA), at 100 μM ligand concentration.

**Table 2:** Kinetic and quality parameters determined by GCI for the interaction of compounds **1**–**3** with *Mab*-SaS.

Code	k_on_ (M^−1^s^−1^)	k_off_ (s^−1^)	K_D_ (uM)	χ^2^
**1**	1.54 ± 0.05 × 10^1^	9.77 ± 0.12 × 10^−5^	6.11 ± 0.16	0.86 ± 0.05
**2**	2.44 ± 0.62 × 10^2^	3.03 ± 0.09 × 10^−3^	12.65 ± 3.32	0.53 ± 0.08
**3**	1.00 ± 0.05 × 10^2^	6.75 ± 0.61 × 10^−3^	67.50 ± 9.51	0.91 ± 0.07

## Supplementary Materials

The following supporting information can be downloaded at: https://www.mdpi.com/article/10.3390/pharmaceutics15020502/s1. Analytical data, Figure S1: Enzymatic activity of *Mab*-SaS as a function of MgCl_2_ concentration; Figure S2: Steady state kinetics analysis of *Mab*-SaS towards chorismate.
