# Supplementary material for: Targeting Siderophore-Mediated Iron Uptake in M. abscessus: A New Strategy to Limit the Virulence of Non-Tuberculous Mycobacteria
Source: Pharmaceutics. 2023 Feb 2;15(2):502. doi: 10.3390/pharmaceutics15020502 (PMC9966845; doi:10.3390/pharmaceutics15020502)
Supplement: Supplementary file 1 [file pharmaceutics-15-00502-s001.zip › pharmaceutics-2147103-supplementary.pdf]

Supplementary Material

Targeting Siderophore-Mediated Iron Uptake in *M. abscessus*: A New Strategy to Limit the Virulence of Non-Tuberculous Mycobacteria

Matteo Mori [1,†], Giovanni Stelitano [2,†], Giulia Cazzaniga [1], Arianna Gelain [1], Andrea Tresoldi [1], Mario Cocorullo [2], Martina Roversi [2], Laurent R. Chiarelli [2], Martina Tomaiuolo [3], Pietro Del Re [4], Giuseppe F. Mangiatordi [4], Anna Griego [1,5], Loris Rizzello [1,5], Alberto Cassetta [3], Sonia Covaceuszach [3,*], Stefania Villa [1,*] and Fiorella Meneghetti [1]

Table of contents

1. Analytical data	S2
2. Figure S1	S3
3. Figure S2	S3

Analytical data

5-(2,4-Bis(trifluoromethyl)2-phenyl)furan-2-carboxylic acid (1)

The compound was synthesized according to a previously published procedure [16].

Aspect: Light brown solid. Yield: 56%. TLC (dichloromethane – methanol 7:3): R_f = 0.57. Mp: 155.7 °C.

^{1}H NMR (300 MHz, DMSO-*d*$_6$) δ (ppm): 13.4-13.2 (bs exch. D_2O, 1H, COOH), 8.17-8.15 (m, 2H, H_8, H_{10}), 8.65 (d, 1H, *J* = 8.2 Hz, H_{11}), 7.36 (d, 1H, *J* = 3.7 Hz, H_3); 7.11 (d, 1H, *J* = 3.7 Hz, H_4).

^{13}C NMR (75 MHz, DMSO-*d*$_6$) δ (ppm): 159.43, 151.77, 146.69, 132.39, 131.98, 130.73-130.24-129.84-129.39 (q), 130.22, 129.39-125.41-121.81-118.19 (q), 128.78-125.17-121.54-117.91 (q), 127.54-127.11-126.68-126.25(q), 124.39,119.55, 114.16.

FTIR (KBr) ν cm^{-1}: 3430, 3180, 3139, 2963, 2918, 2851, 1684, 1628, 1591, 1532, 1490, 1434, 1347, 1267, 1097, 816.

HRMS (ESI-QTOF) *m*/*z*: $C_{13}H_6F_6O_3$ calcd. 323.0148, found 323.0151 [M-H]$^-$.

5-(2-Amino-4-nitrophenyl)furan-2-carboxylic acid (2)

The compound was synthesized according to a previously published procedure [16].

Aspect: orange solid. Yield: 40%. TLC (dichloromethane – methanol 7:3): R_f = 0.52. Mp: 228.5 °C.

^{1}H NMR (300 MHz, DMSO-*d*$_6$) δ (ppm): 13.4-13.2 (bs exch D_2O, 1H, COOH), 7.78-7.73 (m, 2H, H8, H_{11}), 7.46 (dd, 1H, *J* = 8.5, 2.1 Hz, H_{10}), 7.39 (d, 1H, *J* = 3.6 Hz, H_3), 7.18 (d, 1H, *J* = 3.6 Hz, H_4), 6.0 (bs exch D_2O, 2H, NH_2).

^{13}C NMR (75 MHz, DMSO-*d*$_6$) δ (ppm): 159.99, 154.52, 148.65, 146.78, 144.95, 129.17, 120.27, 118.95, 111.94, 111.58, 111.50.

FTIR (KBr) ν cm^{-1}: 3512, 3404, 3120, 2956, 1682, 1633, 1585, 1576, 1508, 1480, 1344, 1314, 1256, 1172, 1040, 1032, 816, 800, 745.

HRMS (ESI-QTOF) *m*/*z*: $C_{11}H_8N_2O_5$ calcd. 247.0360, found 247.0361 [M-H]$^-$.

5-(3,5-Bis(trifluoromethyl)phenyl)furan-2-carboxylic acid (3)

The compound was synthesized according to a previously published procedure [18].

Aspect: white solid. Yield: 91%. TLC (dichloromethane – methanol 7:3): R_f = 0.39. Mp: 168.0 °C.

^{1}H NMR (300 MHz, DMSO-*d*$_6$) δ (ppm): 13.65-13.20 (broad s. exch. D_2O, 1H, COOH), 8.41-8.36 (m, 2H, H_7, H_{11}), 8.12-8.07 (m, 1H, H_9), 7.59 (d, *J* = 3.7 Hz, 1H, H_3), 7.38 (d, *J* = 3.7 Hz, 1H, H_4).

^{13}C NMR (75 MHz, DMSO-*d*$_6$) δ (ppm): 159.48 (COOH), 153.19 (C5), 146.03 (C2), 132.34-131.90-131.46-131.03 (q, C8), 132.00 (C6), 128.98-125.36-121.74-118.12 (q, CF3), 125.00 (C11), 122.14 (C9), 120.14 (C3), 111.73 (C4).

FTIR (KBr) ν cm^{-1}: 2960, 2925, 2855, 1689, 1621, 1591, 1526, 1455, 1420, 1366, 1278, 1161, 1124, 1081, 1027, 896.

HRMS (ESI-QTOF) *m*/*z*: calcd. for $C_{13}H_6F_6O_6$ 324.18, found 323.16 [M-H]$^-$.

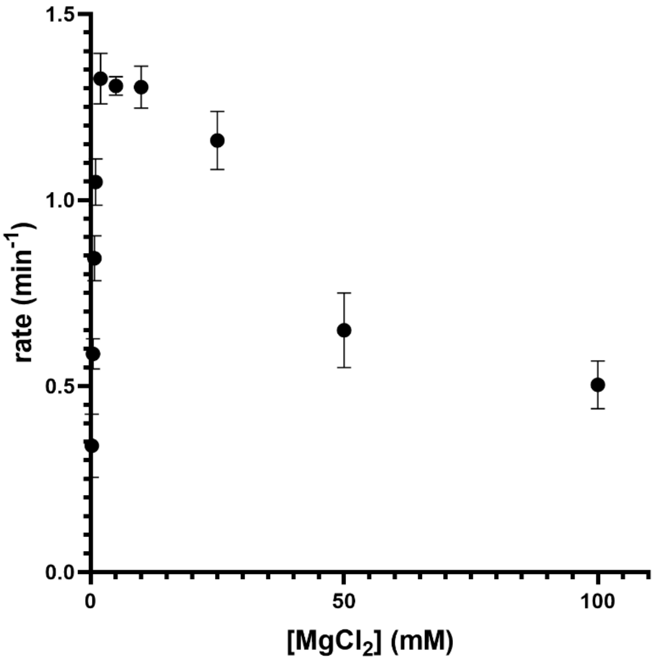

Figure S1. Enzymatic activity of *Mab*-SaS as a function of $MgCl_2$ concentration.

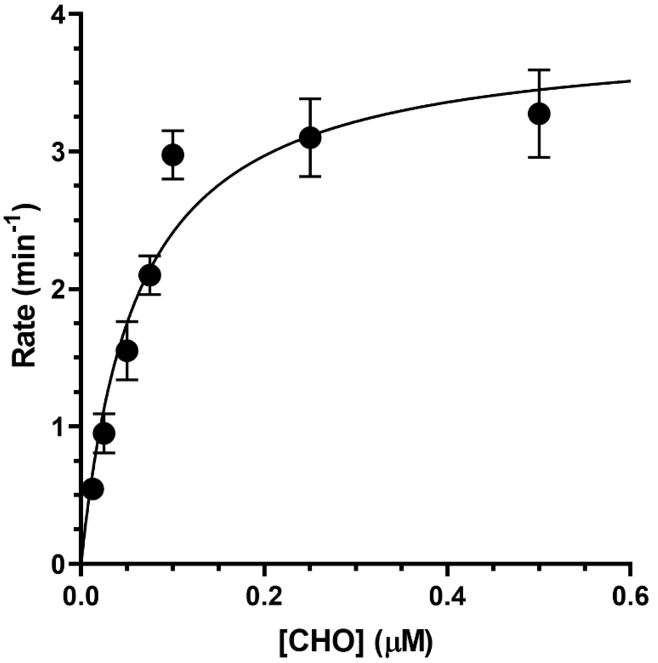

Figure S2. Steady state kinetics analysis towards CHO of *Mab*-SaS.
